# Associations between urinary arsenic and vitamin D deficiency: a cross-sectional analysis of NHANES 2011–2018

**DOI:** 10.1186/s41043-025-01235-0

**Published:** 2026-05-28

**Authors:** Meiling Zhou, Yiyun Liu, Wenqi Liu, Yunwei Zhang

**Affiliations:** 1 Suining Center for Disease Control and Prevention, Suining, Sichuan 629000 China; 2 School of Public Health, Nanjing Medical University, Nanjing, Jiangsu 211166 China; 3 Department of Occupational and Environment Health, Xiangya School of Public Health, Central South University, Changsha, 410078 China; 4 Department of Neurology, Suining Central Hospital, Suining, Sichuan 629000 China

**Keywords:** Urinary arsenic, Dimethylarsinate, Environmental exposure, Vitamin d deficiency, Dose–response, Stroke survivors, Sleep disorders

## Abstract

Arsenic exposure and vitamin D deficiency independently contribute to adverse neurological and systemic outcomes, yet their interplay in the general population has not been thoroughly characterized. We investigated the association between urinary arsenic (total arsenic and dimethylarsinate [DMA]) and vitamin D deficiency and explored how this relationship varies by demographic, behavioral, laboratory, dietary, and neurological comorbidity factors. Using data from 6,148 U.S. adults aged ≥ 18 years in NHANES 2011–2018, we estimated odds ratios (ORs) for deficiency per interquartile‑range increase in each arsenic measure using survey-weighted logistic regression. After full adjustment for demographics, lifestyle, dietary, and clinical factors, the ORs for total urinary arsenic and DMA were 1.30 (95% CI 1.16, 1.45) and 1.50 (95% CI 1.27, 1.78), respectively. Associations were strongest among women; adults aged < 60 years; Non‑Hispanic, highly educated, underweight individuals; as well as current smokers and drinkers. In contrast, participants with stroke or sleep disorders exhibited inverse associations, likely due to routine clinical vitamin D supplementation. These findings demonstrate a robust, dose‑dependent association between arsenic exposure and vitamin D deficiency in U.S. adults. They suggest the importance of integrated public health strategies—combining arsenic mitigation with targeted vitamin D strategies—and highlight the need for longitudinal and interventional studies to establish causality and to determine whether optimizing vitamin D status might potentially modify the adverse health effects of arsenic.

**Supplementary Information:**

The online version contains supplementary material available at 10.1186/s41043-025-01235-0.

## Introduction

Arsenic pollution is a pressing global issue affecting nearly a hundred countries worldwide, with human exposure occurring through occupational, dietary, and environmental routes [1–4]. As a type 1 carcinogen, arsenic exposure is linked to a spectrum of carcinogenic and non-carcinogenic diseases, including cardiovascular disease, diabetes, and cancers [5–12]. Beyond its well-established systemic impacts, emerging evidence highlights arsenic’s neurotoxic effects, particularly its influence on cognitive function and neurological health. Chronic low-dose arsenic exposure has been epidemiologically associated with cognitive deficits in children and neuropathies in adults [13–15].

Vitamin D, a fat-soluble steroid hormone, plays a critical role in maintaining skeletal and neurological health. Vitamin D deficiency is a major public health concern affecting over one billion people globally, and recent studies have linked it to neurodegenerative disorders such as Alzheimer’s and Parkinson’s disease [16–18]. Vitamin D’s neuroprotective effects, mediated by its anti-inflammatory and antioxidant pathways, underscore its importance as a potentially modifiable factor in neurological health [19–22].

A growing body of evidence suggests a complex interaction between arsenic exposure and vitamin D metabolism. Arsenic may disrupt vitamin D homeostasis by impairing hepatic and renal activation pathways, while vitamin D deficiency potentially exacerbates arsenic-induced oxidative stress and neuroinflammation [23–25]. However, despite these plausible mechanisms, population-based data characterizing the relationship between urinary arsenic and vitamin D status remain scarce. To address this gap, this study utilized data from NHANES 2011–2018 to investigate the association between urinary arsenic (total arsenic and dimethylarsinate [DMA]) and vitamin D deficiency. Specifically, the primary objective of this study was to examine the association between urinary arsenic concentrations and vitamin D deficiency, and to explore potential variations across different demographic and clinical subgroups.

## Materials and methods

### Study population

**Fig. 1:**
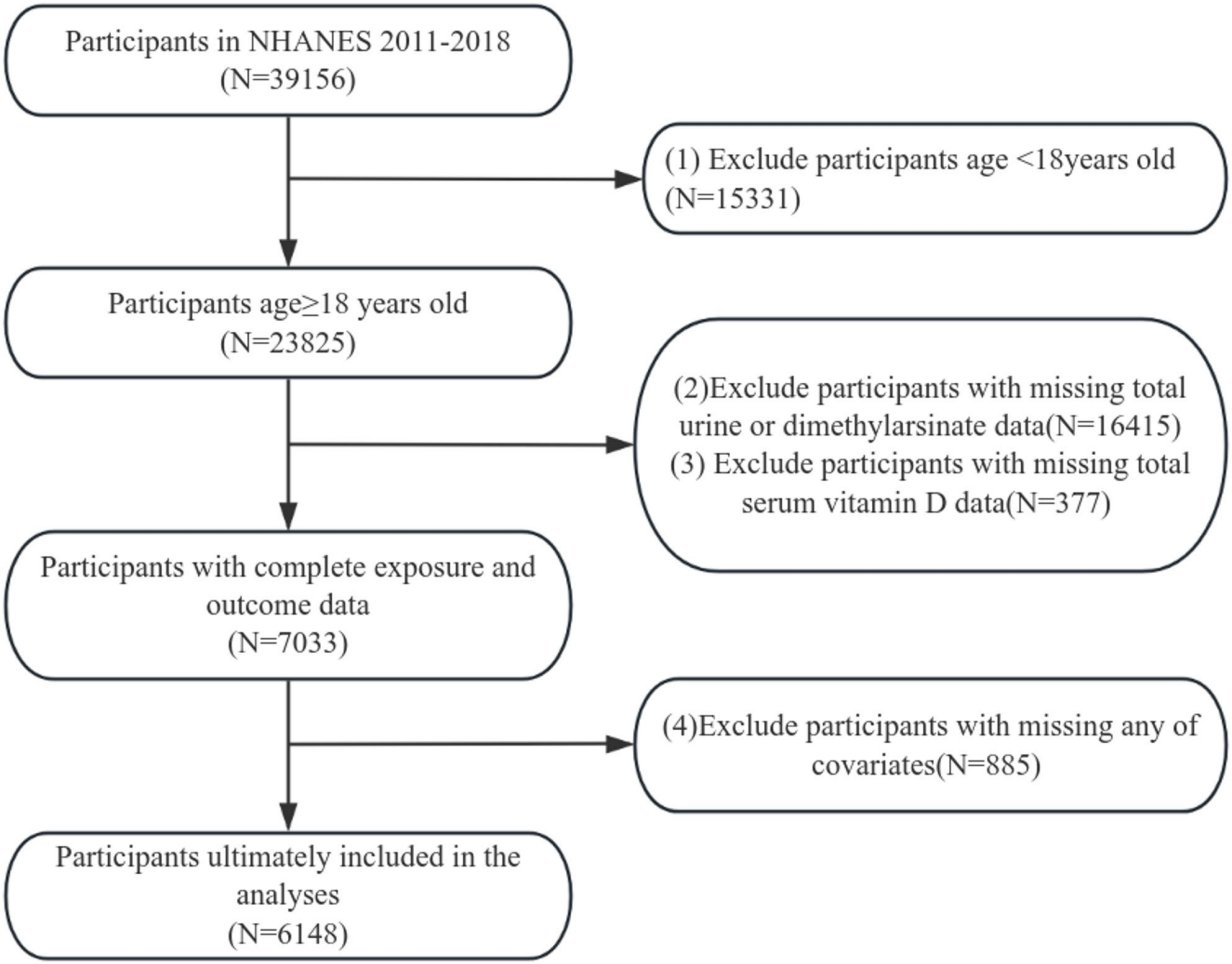
Flowchart showing the selection of the studied population

This study used data from the NHANES database in the United States from 2011 to 2018. The initial total sample size was 39,156. The population of this study is adults, excluding minors under the age of 18, with a remaining sample size of 23,825. Subsequently, consistent with the NHANES sampling strategy where urinary metals are quantified in a random one-third subsample, we excluded participants without urinary arsenic or serum vitamin D measurements, resulting in a sample size of 7033. Finally, 885 missing variable values of interest were removed. The final sample size included in this study was 6148 (Fig. 1). According to the requirements of the Institutional Review Committee of the National Health Statistics Center, NHANES participants provided written informed consent forms.

### Urinary arsenic assay

According to the collection method recorded in the NHANES database, participants collected on-site urine samples at the examination center. In ELAN^®^ DRC Plus or ELAN^®^ Total arsenic in urine was determined by inductively coupled plasma dynamic reactive cell mass spectrometry (ICP-DRC-MS) using a DR™ II ICP-MS instrument (PerkinElmer SCIEX, Concord, on, Canada). Urine samples are stored in pre screened containers. To prevent the interconversion of arsenic species, urine samples are transported or stored at a temperature of ≤ -70℃. Furthermore, arsenic analysis is performed within 3 weeks after collection. The detection limit for total arsenic was 0.23 µg/L in 2011–2012; From 2013 to 2016, it’s 0.26 µg/L; It was 0.23 µg/L in 2017 to 2018. 4.4% of participants had total urinary arsenic levels below the detection limit.

The arsenic substances in urine (urinary arsenous acid, urinary arsenic acid, urinary arsenocholine, and urinary monomethylarsonic acid) are measured by high-performance liquid chromatography and inductively coupled plasma dynamic reaction mass spectrometry. For populations exposed to low or moderate levels of arsenic, the detection limits for urinary arsenous acid, urinary arsenic acid, urinary arsenocholine, and urinary monomethylarsonic acid are too high, with 66.66%, 96.88%, 92.23%, and 79.41% of sample participants having levels below the detection limits for urinary arsenous acid, urinary arsenic acid, urinary arsenocholine, and urinary monomethylarsonic acid, respectively. Therefore, levels of urinary arsenous acid, urinary arsenic acid, urinary arsenocholine, and urinary monomethylarsonic acid are not used in our analysis. The detection limit of dimethylarsinate is 1.91 µg/L. The percentage of participants in the study with dimethylarsinate content below the detection limit is 25.76%. For participants with total arsenic and dimethylarsinate levels below the detection limit, the estimated level is equal to the detection limit divided by the square root of 2.

### The determination of vitamin D metabolites

According to the NHANES data collection guidelines, use conventional red top or serum separators Vacutainers™ Collect serum samples. 25 hydroxytotal vitamin D (total 25 OHD) in human serum is quantitatively detected using ultra-high performance liquid chromatography-mass spectrometry (UHPLC-MS/MS). The analyte is typically separated by chromatography on one of three pentafluorophenyl (PFP) columns (ThermoScientific, Waltham, MA, USA, Hypersil GOLD PFP 2.1 × 100 mm, 1.9 μm particle size column, Phenomenex, Torrance, CA, USA, Kinetex PFP 2.1 × 100 mm, 1.7 μm, or SigmaAldrich, St. Louis, MO, USA, Ascentis Express F5, 2.1 × 150 mm, 2.7 μm). Detection is performed using a triple quadrupole tandem mass spectrometer (Thermo TSQ Vantagesystem) in positive ion mode using atmospheric pressure chemical ionization. Quantification is achieved by comparing the response rate of an unknown substance with the response rate of a known amount of analyte in a calibration solution. The response ratio is based on the peak area of the analyte divided by the peak region of the internal standard [26].

### Other covariates

The basic information survey of participants includes general demographic characteristics and life behaviors, as well as general demographic characteristics such as gender, age, race, and education level. General behavioral characteristics include drinking and smoking. Diseases related to the nervous system, such as sleep disorders and stroke.The above information is based on the self-report of the participants. In addition, body mass index (BMI) is calculated by dividing body weight (kg) by the square of height (meters). Using Beckman UniCel ^®^ DxC 800 Synchron and Beckman UniCel ^®^ DxC 660i Synchron Access clinical system measures cholesterol. Measure serum cotinine by isotope dilution high performance liquid chromatography/atmospheric pressure chemical ionization tandem mass spectrometry (ID HPLC-APCI MS/MS). As for dietary intake, the current nutritional assessment section of NHANES includes a 24-hour dietary reuptake view for participants of all age groups. The dietary recall interview is conducted by trained dietary interviewers who are fluent in Spanish and English. The interview will take place in a private room at the mobile exam center. Each MEC dietary interview room includes a set of standard measurement guidelines.

### Statistical analysis

All statistical analyses were performed using the survey package in R version 4.5.1 and SPSS (v.25.0). To ensure national representativeness, all analyses accounted for the complex survey design by incorporating sampling strata (SDMVSTRA), primary sampling units (SDMVPSU), and constructed multi-cycle subsample weights. Statistical significance was determined using two-sided tests with a significance level of α < 0.05. Missing values for covariates with a missing rate of < 1% were imputed using the mean for continuous variables (e.g., BMI) and the mode for categorical variables (e.g., smoking status). Since the distribution of total urinary arsenic and dimethylarsinate concentrations was skewed to the right, we performed logarithmic transformation on the data. Due to the non-normal distribution, median and interquartile range (IQR) were used to describe these samples. The relationship between participant characteristics and urinary arsenic concentration (total urinary arsenic and dimethylarsinate) was evaluated using the Wilcoxon rank sum test or Kruskal-Wallis test. We modeled urinary arsenic as both a continuous variable and a categorical variable (tertiles). For continuous analyses, we calculated ORs per Interquartile Range (IQR) increase. Additionally, to evaluate the sensitivity of the association to different exposure magnitudes, we estimated ORs comparing the 80th vs. 20th percentiles (representing a substantial “high vs. low” exposure difference) and the 70th vs. 30th percentiles (representing a moderate difference typical of the central population). To assess non-linearity, we used restricted cubic splines with knots at the 5th, 50th, and 95th percentiles. Three adjustment models were used: the first model was unadjusted; the second model was adjusted for gender, age, race and nationality, education level, body mass index, sleep disorder, stroke, drinking, and smoking; while the third model further corrected for serum cotinine, cholesterol, and dietary intake (carbohydrate, fat, dietary fiber, and protein) to rigorously control for potential confounding by dietary sources of arsenic and metabolic factors. Subgroup analyses were performed to evaluate the consistency of the results across strata defined by gender, age, race and ethnicity, education, body mass index, diabetes, smoking status, and alcohol consumption. Given the exploratory nature of these analyses, we did not apply corrections for multiple comparisons to avoid inflating Type II errors; instead, we relied on interaction tests to assess heterogeneity. Consequently, these subgroup findings should be interpreted as hypothesis-generating.

## Results

Table 1 presents the characteristics of the 6148 participants included in the study. The study population was approximately evenly distributed by gender (50.5% men and 49.5% women) and spanned all adult age groups. The major of participants was Non-Hispanic (60.5%), and 78.6% had attained a high school education or above. Regarding body weight, 38.6% of individuals were classified as obese (BMI ≥ 30). Lifestyle comparisons showed that 76.1% of the participants reported alcohol consumption, while 41.8% had a history of smoking. In terms of comorbidities, 27.0% reported sleep disorders, and 3.8% reported a history of stroke. Based on the standard clinical threshold established by the Institute of Medicine (IOM) (serum 25(OH)D < 50 nmol/L), the overall weighted prevalence of vitamin D deficiency in the study population was 31.8%. The overall median concentrations of total urinary arsenic and dimethylarsinate (DMA) were 6.58 µg/L (IQR: 3.38–14.03) and 3.32 µg/L (IQR: 1.34–5.80), respectively. Univariate analyses indicated significant variations in arsenic levels across demographic groups. Specifically, median total urine arsenic concentrations were significantly higher in men, older adults (≥ 60 years), individuals of other races, and those with higher education levels or lower BMI (*P* <0.05). Regarding comorbidities, participants without sleep disorders or stroke exhibited higher median urinary arsenic levels. Furthermore, participants with vitamin D deficiency had significantly higher urinary arsenic levels (6.89 µg/L, *P* = 0.002). In addition, participants with carbohydrate intake < 189.6 g, protein intake greater than 89.9 g, fat intake less than 58.8 g, and dietary fiber intake between 11.9 and 19.0 g have higher concentrations of total urinary arsenic. Detailed associations between dietary intake (carbohydrate, protein, fat, and dietary fiber) and urinary arsenic concentrations are presented in Supplementary Table S1. Generally, distinct patterns of arsenic exposure were observed across different quartiles of macronutrient intake. Of the 7,033 eligible participants, 885 were excluded primarily due to missing data on dietary intake (*N* = 575) and alcohol consumption (*N* = 310). Comparisons between the initial population (*N* = 7,033) and the final analytical sample (*N* = 6,148) are presented in Supplementary Table S2. Importantly, no significant differences were observed in vitamin D status (*P* > 0.05). While statistically significant differences were noted for race and urinary arsenic levels due to the large sample size, the absolute magnitudes of these differences were small (e.g., total arsenic difference < 0.3 µg/L), suggesting that the analytical sample remains representative.

**Table 1:** Urine arsenic concentrations by participant characteristics

Characteristics	No. (%)	Total Urine Arsenic median (IQR), µg/L	Dimethylarsinate median (IQR), µg/L	*P* Value^a^	*P* Value^b^
Overall	6148	6.58 (3.38–14.03)	3.32 (1.34–5.80)		
Sex				< 0.001	< 0.001
Men	3105 (50.5)	7.25 (3.76–14.99)	3.61 (1.99–6.02)		
Women	3043 (49.5)	6.00 (2.95–13.16)	3.03 (1.35–5.54)		
Age, y				< 0.001	0.641
18–39	2288 (37.2)	6.22(3.36–12.44)	3.32 (1.35–5.82)		
40–59	1894 (30.8)	6.41 (3.18–13.80)	3.24 (1.35–5.80)		
≥ 60	1966 (32.0)	7.37 (3.71–16.39)	3.39 (1.35–5.80)		
Race				< 0.001	< 0.001
Chicano	873 (14.2)	6.14 (3.21–10.88)	3.35 (1.91–5.26)		
Other Hispanics	635 (10.3)	7.45 (4.15–15.61)	4.13(2.32–7.36)		
Non-Hispanic	3717 (60.5)	5.97 (3.13–12.73)	2.95 (1.35–4.94)		
Other Races	923 (15.0)	10.55 (4.36–26.67)	5.07 (2.38–9.61)		
Education status				0.01	0.08
< High school	1313 (21.4)	6.24 (3.27–13.29)	3.30 (1.35–5.81)		
High school	1448 (23.5)	6.28 (3.36–12.95)	3.15 (1.35–5.50)		
> High school	3387 (55.1)	6.87 (3.43–15.05)	3.40 (1.35–5.94)		
BMI				0.723	0.979
< 25	1784 (29.0)	6.63 (3.15–15.91)	3.23 (1.35–5.97)		
25-<30	1989 (32.4)	6.58 (3.43–13.70)	3.36 (1.35–5.81)		
≥ 30	2375 (38.6)	6.48 (3.51–13.36)	3.33 (1.94–5.66)		
Drinking				0.358	0.363
Yes	4676 (76.1)	6.60 (3.47–13.86)	3.32 (1.35–5.75)		
No	1472 (23.9)	6.43 (3.02–14.24)	3.32 (1.35–5.98)		
Stroke				0.397	0.078
Yes	231 (3.8)	6.41 (3.24–12.88)	3.09 (1.35–5.23)		
No	5917 (96.2)	6.58 (3.38–14.13)	3.32 (1.35–5.83)		
Sleep disorder				0.017	< 0.001
Yes	1659 (27.0)	6.21 (3.11–13.13)	3.05 (1.35–5.34)		
No	4489 (73.0)	6.72 (3.49–14.45)	3.43 (1.80–5.98)		
Vitamin D deficiency				0.002	< 0.001
Yes	1954 (31.8)	6.89 (3.85–14.37)	3.62 (2.03–6.27)		
No	4194 (68.2)	6.40 (3.22–13.75)	3.19 (1.35–5.58)		
Smoking				< 0.001	< 0.001
Yes	2572 (41.8)	6.17 (3.23–13.05)	3.15 (1.35–5.47)		
No	357 (58.2)	6.85 (3.52–14.88)	3.45 (1.84–6.02)		
Serum cotinine (ng/mL)				< 0.001	< 0.001
< 0.016	2033 (33.1)	6.84 (3.47–13.69)	3.37 (1.35–5.85)		
0.0164–0.207	2067 (33.6)	7.04 (3.59–15.92)	3.50 (1.92–6.25)		
≥ 0.207	2048 (33.3)	6.01 (3.18–12.73)	3.10 (1.35–5.34)		
Cholesterol (mmol/L)				0.426	0.012
< 4.4	2024 (32.9)	6.31 (3.31–13.72)	3.18 (1.35–5.53)		
4.4–5.3	2066 (33.6)	6.87 (3.46–14.20)	3.40 (1.87–6.05)		
≥ 5.3	2058 (33.5)	6.55 (3.38–14.33)	3.40 (1.35–5.82)		

IQR, Interquartile Range; BMI, Body Mass Index

^a^ *P* value based on log-transformed total urine arsenic concentrations

^*b*^ *P* value based on log-transformed dimethylarsinate concentrations

Models were adjusted as described in the Methods. *P* value < 0.05 were considered statistically significant

In our study, without adjusting for any confounding factors, the ORs for vitamin D deficiency in participants with total urinary arsenic and dimethylarsinate are 1.18 (95% CI 1.06, 1.32) and 1.43 (95% CI 1.23, 1.67), respectively. The ORs for vitamin D deficiency in participants at the 70th and 30th percentiles of total urine arsenic and dimethylarsinate are 1.20 (95% CI 1.08, 1.34) and 1.39 (95% CI 1.20, 1.60), separately. The ORs for vitamin D deficiency in participants at the 80th and 20th percentiles of total urine arsenic and dimethylarsinate are 1.21 (95% CI 1.08, 1.36) and 1.37 (95% CI 1.18, 1.58), respectively (Table 2; model 1). In addition, after adjusting for gender, age, race, education level, BMI, smoking, drinking, sleep disorder and stroke, the ORs values of total urinary arsenic and dimethylarsinate in vitamin d deficient participants are 1.27 (95% CI 1.13, 1.42) and 1.45 (95% CI 1.23, 1.71), respectively. The ORs for vitamin D deficiency in participants at the 70th and 30th percentiles of total urine arsenic and dimethylarsinate are 1.27 (95% CI 1.13, 1.44) and 1.34 (95% CI 1.15, 1.56), separately. The ORs for vitamin D deficiency in participants at the 80th and 20th percentiles of total urine arsenic and dimethylarsinate are 1.29 (95% CI 1.13, 1.46) and 1.33 (95% CI 1.14, 1.56), respectively (Table 2; model 2). Furthermore, we further adjust the laboratory test data (Serum cotinine and cholesterol) and dietary related data (carbohydrate, protein, fat and dietary fiber) based on Model 2, and the results showed that the ORs values of total urine arsenic and dimethylarsinate for participants with vitamin D deficiency are 1.30 (95% CI 1.16, 1.45) and 1.50 (95% CI 1.27, 1.78), respectively. The ORs for vitamin D deficiency in participants at the 70th and 30th percentiles of total urine arsenic and dimethylarsinate are 1.32 (95% CI 1.17, 1.49) and 1.39 (95% CI 1.19, 1.63), separately. The ORs for vitamin D deficiency in participants at the 80th and 20th percentiles of total urine arsenic and dimethylarsinate are 1.34 (95% CI 1.17, 1.52) and 1.39 (95% CI 1.18, 1.64), respectively (Table 2; model 3).

**Table 2:** Odds ratio of vitamin D deficiency by urine arsenic concentrations

	With Vitamin D deficiency/without Vitamin D deficiency	70th vs. 30th percentile	80th vs. 20th percentile
Total Urine Arsenic (µg/L)	6.89/6.40	12.50/3.80	19.38/2.75
Model 1^a^	1.18 (1.06–1.32)	1.20 (1.08–1.34)	1.21 (1.08–1.36)
Model 2^b^	1.27 (1.13–1.42)	1.27 (1.13–1.44)	1.29 (1.13–1.46)
Model 3^c^	1.30 (1.16–1.45)	1.32 (1.17–1.49)	1.34 (1.17–1.52)
Dimethylarsinate (µg/L)	3.62/3.19	5.57/2.01	6.71/1.35
Model 1^a^	1.43 (1.23–1.67)	1.39 (1.20–1.60)	1.37 (1.18–1.58)
Model 2^b^	1.45 (1.23–1.71)	1.34 (1.15–1.56)	1.33 (1.14–1.56)
Model 3^c^	1.50 (1.27–1.78)	1.39 (1.19–1.63)	1.39 (1.18–1.64)

CI, Confidence Interval

^a^Model 1 is shown as odds ratio (95% CI); Unadjusted estimates

^b^Model 2 is shown as odds ratio (95% CI); further adjusted for sex, age, race and ethnicity and education status. body mass index (calculated as weight in kilograms divided by height in meters squared), drinking, smoking, sleep disorder and stroke

^c^Model 3 is shown as odds ratio (95% CI); further adjusted for Serum cotinine, cholesterol and dietary intake (carbohydrate, protein, fat and dietary fiber)

To further characterize the relationship between urinary arsenic and vitamin D deficiency, we performed tertile analyses for total urinary arsenic and dimethylarsinate to evaluate dose-response trends at different urinary total arsenic and dimethyl arsenate concentrations. When adjusting for confounding factors (Model 1), compare to the first group (with the lowest urine arsenic concentration), the other two groups of participants with vitamin D deficiency had an increase in ORs greater than 1, the difference is statistically significant (Table 3). After adjusting for confounding factors (Model 2 and Model 3), as the total urine arsenic concentration increased, the ORs of participants with vitamin D deficiency increased, and the difference was statistically significant (Table 3). Additionally, In the three models, as the dimethylarsinate concentration increase, the ORs of participants with vitamin D deficiency increase, the difference is statistically significant (Table 3).

**Table 3:** Odds ratios of vitamin D deficiency by urine arsenic concentrations

	Tertile 1	Tertile 2	Tertile 3	*P* Value for Trend^a^
Total Urine Arsenic (µg/L)	< 4.29	4.29 to10.67	> 10.67	
With Vitamin D deficiency/without Vitamin D deficiency, No.	589/1457	685/1367	680/1370	
Model 1^b^	1[Reference]	1.24 (1.09–1.42)	1.23 (1.08–1.40)	0.002
Model 2^c^	1[Reference]	1.22 (1.06–1.40)	1.32 (1.15–1.52)	< 0.001
Model 3^d^	1[Reference]	1.24 (1.08–1.42)	1.36 (1.18–1.57)	< 0.001
Dimethylarsinate (µg/L)	< 2.27	2.27 to4.67	> 4.67	
With Vitamin D deficiency/without Vitamin D deficiency, No.	576/1469	652/1398	726/1327	
Model 1^b^	1[Reference]	1.19 (1.04–1.36)	1.40 (1.22–1.59)	< 0.001
Model 2^c^	1[Reference]	1.13 (0.99–1.30)	1.33 (1.15–1.53)	< 0.001
Model 3^d^	1[Reference]	1.14 (0.99–1.31)	1.36 (1.18–1.57)	< 0.001

CI, Confidence Interval

^a^ *P* value for trend based on log-transformed total urine arsenic and dimethylarsinate concentrations

^b^ Model 1 is shown as odds ratio (95% CI); Unadjusted estimates

^c^ Model 2 is shown as odds ratio (95% CI); further adjusted for sex, age, race and ethnicity and education status. body mass index (calculated as weight in kilograms divided by height in meters squared), drinking and smoking, sleep disorder, stroke

^d^ Model 3 is shown as odds ratio (95% CI); further adjusted for Serum cotinine, cholesterol and dietary intake (carbohydrate, protein, fat and dietary fiber)

Models were adjusted as described in the Methods. *P* value < 0.05 were considered statistically significant

**Fig. 2:**
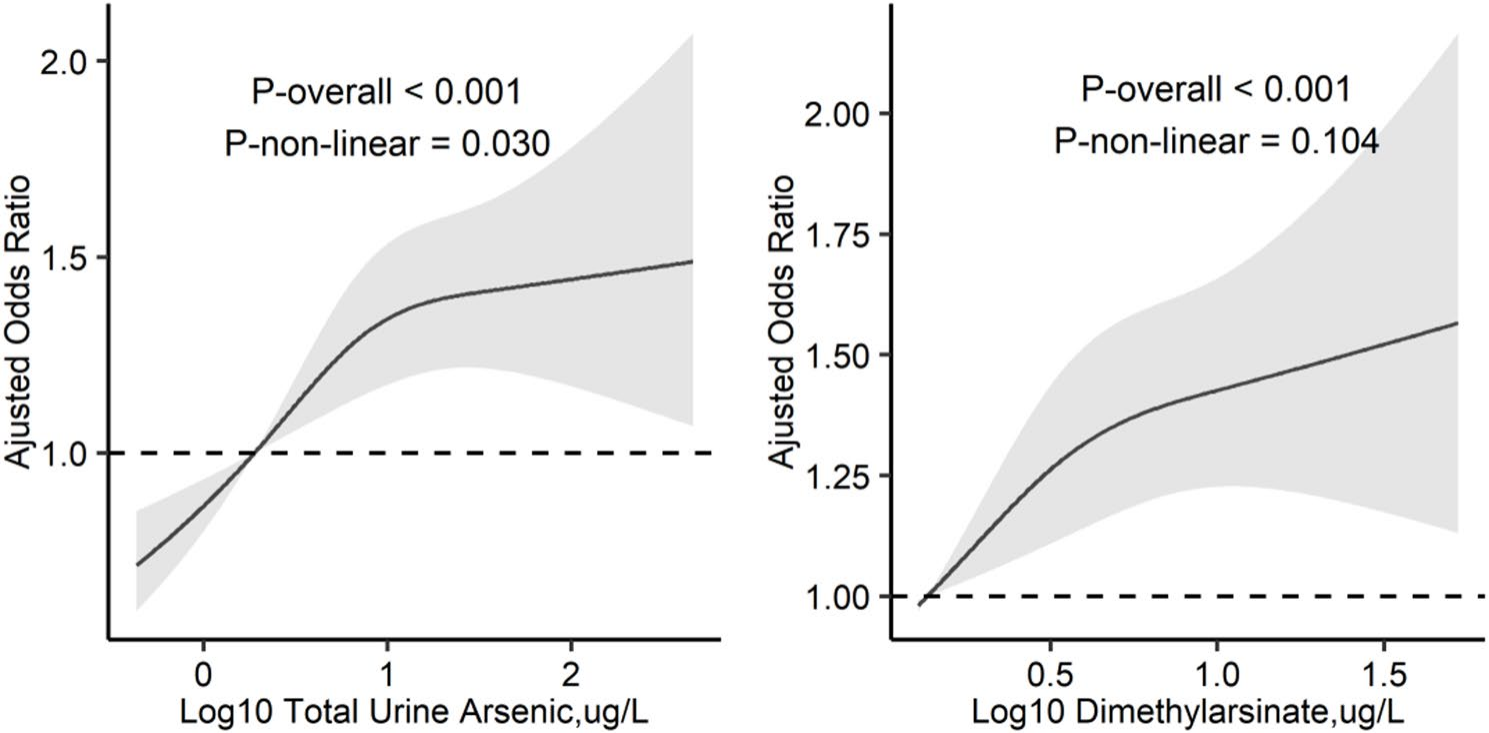
Odds Ratio of Vitamin D deficiency by Urine Arsenic Concentrations. Lines represent adjusted odds ratios based on restricted cubic splines (RCS) for log-transformed arsenic concentrations with knots at 5th, 50th, and 95th percentiles. The reference value was set at the 10th percentile of each arsenical distribution. Odds ratios were adjusted sex, age, race and ethnicity and education status. body mass index, drinking and smoking, sleep disorder, stroke, serum cotinine, cholesterol and dietary intake (carbohydrate, protein, fat and dietary fiber)

To characterize the dose-response relationship, we employed restricted cubic splines (Fig. 2). After adjusting for confounding factors, we observed distinct patterns for the two biomarkers. Total urinary arsenic exhibited a significant non-linear association (*P* = 0.030), characterized by a sharp increase in the odds of deficiency at lower concentrations which tended to plateau at higher levels. In contrast, the relationship for dimethylarsinate (DMA) was approximately linear (*P* = 0.104), showing a consistent, monotonic upward trend in risk as concentrations increased.

**Fig. 3:**
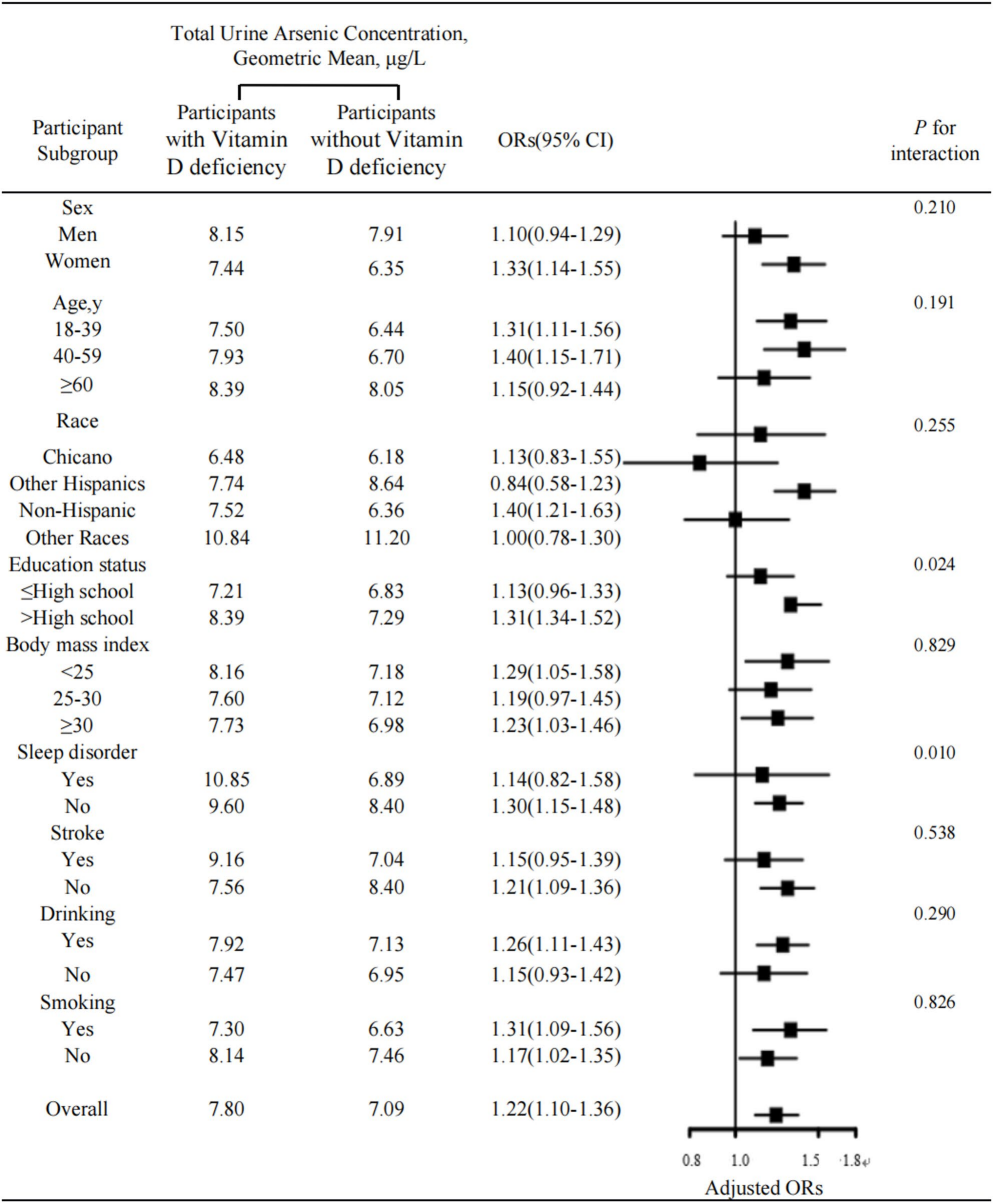
Urine Arsenic Concentrations Comparing Participants With Vitamin D deficiency vs. Participants Without. ^a^ Ratio (95% confidence interval) is also adjusted for serum cotinine, cholesterol and dietary intake (carbohydrate, protein, fat and dietary fiber). ^b^ Body mass index is calculated as weight in kilograms divided by height in meters squared

**Fig. 4:**
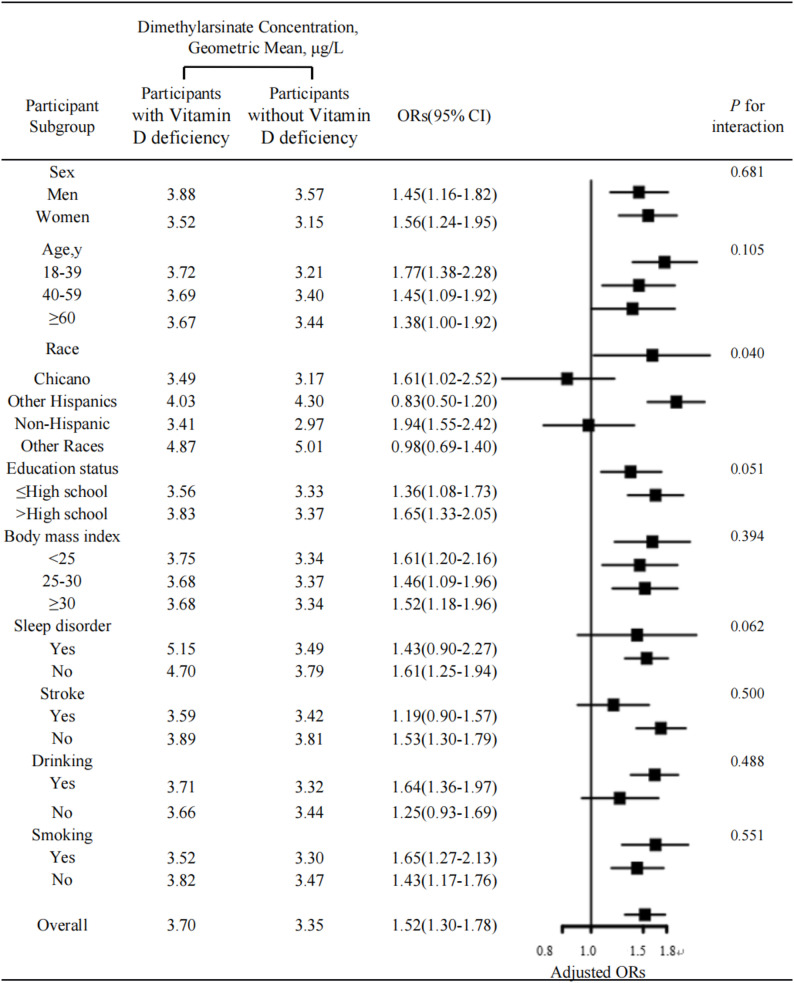
Dimethylarsinate Concentrations Comparing Participants With Vitamin D deficiency vs. Participants Without. ^a^ Ratio (95% confidence interval) is also adjusted for serum cotinine, cholesterol and dietary intake (carbohydrate, protein, fat and dietary fiber). ^b^ Body mass index is calculated as weight in kilograms divided by height in meters squared

After adjusting for serum cotinine, cholesterol and diet related factors (carbohydrate, protein, fat and dietary fiber), the positive correlation between total urine arsenic and vitamin D deficiency is consistent in most subgroups. It’s more obvious in women, adults aged 40–59 years, Non-Hispanic, highly educated individuals, underweight participants (BMI < 25), and current smokers and drinkers, as well as those without sleep disorders or stroke (Fig. 3). Similarly, the association with DMA was also stronger in these subgroups (Fig. 4).

## Discussion

In this large NHANES cohort study, we observed a robust dose–response relationship between urinary arsenic concentrations (total arsenic and DMA) and the prevalent vitamin D deficiency. The consistent increase in deficiency odds across arsenic quartiles, confirmed by monotonic spline curve analyses, establishes a positive association between arsenic exposure and vitamin D status in the general U.S. adult population.

Possible biological mechanisms underlying these findings involve multiple metabolic pathways. Arsenic and related heavy metals may act as nephrotoxins that inhibit the renal 1α-hydroxylase (CYP27B1) enzyme, thereby potentially impairing the conversion of 25-hydroxyvitamin D [25(OH)D] to its biologically active form, 1,25-dihydroxyvitamin D [1,25(OH)_2_D] [27, 28]. Moreover, prior studies indicate that arsenic-induced oxidative stress could disrupt cytochrome P450 enzymes integral to vitamin D metabolism, leading to downregulation of vitamin D-binding proteins and receptors, further diminishing circulating vitamin D levels [23, 29]. Furthermore, this relationship may be bidirectional; emerging evidence suggests that vitamin D receptor (VDR) activation can influence arsenic metabolism and excretion, potentially creating a cycle where deficiency exacerbates toxicity [30].

Notably, our analysis additionally revealed novel effect modification by comorbid conditions. Among participants without a history of stroke or chronic sleep disorders, the positive association between arsenic exposure and vitamin D deficiency was clear and dose-responsive. Conversely, paradoxical inverse associations emerged in individuals with stroke or sleep disorders. While we could not directly quantify supplement intake due to excessive data missingness (> 60%), this counterintuitive finding may be attributable to negative confounding by healthcare interventions. Stroke survivors commonly receive vitamin D and calcium supplementation during rehabilitation, improving vitamin D status [31, 32]. Similarly, patients with sleep disorders often receive vitamin D supplementation due to evidence linking low 25(OH)D levels with impaired sleep quality [33, 34]. In practice, these patients may be more likely to get vitamin D repletion (through diet, sun exposure advice, or supplements) as part of standard care. Therefore, it is plausible that despite high arsenic exposure, these subgroups display artificially elevated 25(OH)D levels due to exogenous supplementation.

Our stratified findings also highlight subgroup heterogeneity. The association appeared stronger among women, adults aged < 60 years, Non-Hispanic individuals, those with higher education, underweight individuals, and current smokers and drinkers, as well as participants without a history of sleep disorders or stroke. Obesity sequesters vitamin D in adipose tissue, reducing its bioavailability [35, 36], while sex and age differences may influence arsenic biotransformation rates or baseline vitamin D levels [37, 38]. Moreover, lifestyle factors such as smoking and alcohol consumption may further exacerbate susceptibility. However, it is important to note that these interpretations regarding subgroup vulnerability are hypothesis-generating. Further mechanistic and longitudinal studies are required to confirm whether these specific physiological traits causally modulate the arsenic–vitamin D interaction.

From a public health perspective, these findings imply that addressing environmental exposure is crucial for nutritional health. Despite regulatory measures, such as the U.S. EPA’s limit of 10 µg/L for drinking water, significant exposure persists, necessitating robust testing, filtration systems, and alternative water supplies [39, 40]. Concurrently, vitamin D deficiency remains prevalent, especially among older adults, individuals with limited sun exposure, and populations with poor dietary intake [38, 41]. Integrating arsenic mitigation efforts such as community education and well water testing with nutrition focused interventions like food fortification and targeted supplementation can yield synergistic benefits [41, 42]. Rather than viewing arsenic toxicity and vitamin D deficiency as separate issues, our data suggest they act as dual burdens. Clinicians, especially those managing patients with risk factors for neurological conditions, should consider the potential dual burden of environmental exposure and nutritional deficiency. Our findings regarding the arsenic-vitamin D link are hypothesis-generating and suggest that incorporating environmental exposure assessments into routine care could provide a more comprehensive risk profile, though clinical trials are needed to determine if such strategies directly improve neurological outcomes.

Despite these insights, some limitations warrant mention. First, the cross-sectional design precludes causal inference, limiting our ability to determine whether arsenic exposure precedes vitamin D deficiency. Second, urinary arsenic and 25(OH)D levels were measured at a single time point. Since urinary arsenic primarily reflects recent exposure, a single measurement may not perfectly capture long-term exposure patterns. Third, we utilized spot urine samples without adjusting for urinary creatinine. While creatinine adjustment is often used to correct for urine dilution, creatinine levels are heavily influenced by muscle mass, which is biologically related to vitamin D status. Consequently, adjusting for creatinine in this context could introduce over-adjustment bias; however, we acknowledge that this approach may not fully account for variations in hydration status. Fourth, self-reported comorbidities, such as stroke and sleep disorders, lack clinical diagnostic confirmation and may be subject to misclassification; however, these data were collected using standardized NHANES protocols intended for population surveillance. Fifth, the exclusion of participants with missing covariates resulted in a complete-case analysis, which may introduce selection bias. However, our sensitivity analysis indicated that while minor statistical differences in arsenic levels were observed—likely due to the large sample size—the distribution of the primary outcome (vitamin D deficiency) remained virtually identical between the initial and analytical samples (*P* > 0.05), suggesting minimal bias. Sixth, we acknowledge that while we discuss our findings in the context of neurological health mechanisms, our dataset did not include direct clinical measures of cognitive function or neurodegeneration. Therefore, implications regarding the prevention of neurological disorders should be interpreted as hypothesis-generating, focusing on the interplay of established risk factors rather than direct clinical outcomes. Finally, despite extensive statistical adjustment, the possibility of residual confounding remains. Specifically, we were unable to adjust for dietary supplement intake in our models because the relevant NHANES data regarding vitamin D and multivitamin use showed excessive missingness (> 60%) in our study sample. Other unmeasured factors, such as specific sun exposure habits or geographic location, may also contribute. Nonetheless, the large sample size, comprehensive covariate adjustment, and validated exposure biomarkers lend robustness to our findings.

Further research is essential to build on these findings. Longitudinal cohort studies could establish the temporal sequence and causality of the arsenic–vitamin D relationship. Advanced causal modeling approaches, such as marginal structural models for time-varying confounders or Mendelian randomization using genetic variants in arsenic metabolism pathways, could clarify causal mechanisms [23]. Improved exposure assessment methods, including biomarkers like toenail arsenic for long-term intake and dietary arsenic speciation, would enhance risk source identification [43]. Finally, intervention studies exploring whether vitamin D supplementation can modify the adverse outcomes associated with arsenic exposure could provide actionable insights into integrated prevention strategies.

## Conclusion

This study demonstrates a robust, dose-dependent association between urinary arsenic exposure and vitamin D deficiency in the U.S. population. These findings highlight the potential interplay between environmental toxicity and nutritional status, suggesting that arsenic exposure may be a significant, under-recognized risk factor for vitamin D deficiency. However, given the cross-sectional design, these insights should be interpreted as hypothesis-generating. While the results point to the potential benefit of integrated strategies—combining arsenic mitigation with nutritional monitoring—longitudinal and interventional studies are a prerequisite to establish causality. Specifically, future research must rigorously evaluate whether optimizing vitamin D status can effectively mitigate the adverse systemic and neurological effects of arsenic exposure before broad public health interventions can be recommended.

## Supplementary Information

Supplementary Material 1

Supplementary Material 2

## Data Availability

Some or all data, models, or code generated or used during the study are available from the corresponding author.
