# Supplementary material for: Associations between urinary arsenic and vitamin D deficiency: a cross-sectional analysis of NHANES 2011–2018
Source: J Health Popul Nutr. 2026 May 28;45:164. doi: 10.1186/s41043-025-01235-0 (PMC13330044; doi:10.1186/s41043-025-01235-0)
Supplement: Supplementary file 1 — Supplementary Material 1 [file 41043_2025_1235_MOESM1_ESM.pdf]

Supplementary Table 1 Urine arsenic concentrations by Participant Characteristics

Characteristics	No. (%)	Total Urine Arsenic median (IQR), μg/L	Dimethylarsinate median (IQR), μg/L	*P* Value[a]	*P* Value[b]
Overall	6148	6.58(3.38-14.03)	3.32(1.34-5.80)		
Dietary intake					
Carbohydrate (g)				0.001	0.347
<189.6	2047(33.3)	6.87(3.41-15.12)	3.31(1.35-5.86)		
189.6-273.2	2053(33.4)	6.82(3.45-14.79)	3.37(1.35-5.89)		
≥273.2	2048(33.3)	6.13(3.30-12.44)	3.29(1.35-5.69)		
Protein (g)				<0.001	0.002
<61.9	2047(33.3)	6.17(3.10-13.14)	3.17(1.35-5.54)		
61.9-89.9	2053(33.4)	6.63(3.38-13.62)	3.28(1.35-5.66)		
≥89.9	2048(33.3)	7.09(3.66-15.33)	3.52(1.35-6.13)		
Fat (g)				0.005	<0.001
<58.8	2047(33.3)	6.93(3.42-15.81)	3.44(1.35-6.44)		
58.8-89.2	2052(33.4)	6.51(3.36-13.79)	3.31(1.35-5.58)		
≥89.2	2049(33.3)	6.34(3.38-12.69)	3.22(1.35-5.44)		
Dietary fiber (g)				0.241	0.642
<11.9	2044(33.2)	6.39(3.28-15.08)	3.22(1.35-5.75)		
11.9-19.0	2053(33.4)	6.86(3.64-13.66)	3.39(1.92-5.81)		
≥19.0	2051(33.4)	6.46(3.24-13.55)	3.36(1.35-5.84)		

Abbreviation: IQR, interquartile range.

[a] *P* value based on log-transformed total urine arsenic concentrations.

[b] *P* value based on log-transformed dimethylarsinate concentrations
