# Supplementary material for: Associations between urinary arsenic and vitamin D deficiency: a cross-sectional analysis of NHANES 2011–2018
Source: J Health Popul Nutr. 2026 May 28;45:164. doi: 10.1186/s41043-025-01235-0 (PMC13330044; doi:10.1186/s41043-025-01235-0)
Supplement: Supplementary file 2 — Supplementary Material 2 [file 41043_2025_1235_MOESM2_ESM.pdf]

Supplementary Table S2. Comparison of characteristics between the initial eligible population and the final analytical population

Characteristics	Initial participants(N=7033)	Included participants(N=6148)	*P* value
Sex			
Men	3473(49.38)	3105(50.5)	0.198
Women	3560(50.62)	3043(49.5)	
Race			
Chicano	1012(14.39)	873(14.2)	0.030
Other Hispanics	743(10.56)	635(10.3)	
Non-Hispanic	4101(58.31)	3717(60.5)	
Other Races	1177(16.74)	923(15.0)	
Education status			0.219
<High school	1464(20.82)	1313(21.4)	
High school	1747(24.84)	1448(23.5)	
>High school	3822(54.34)	3387(55.1)	
Stroke			0.940
Yes	266(3.78)	231(3.8)	
No	6767(96.22)	5917(96.2)	
Sleep disorder			0.475
Yes	1859(26.43)	1659(27.0)	
No	5174(73.57)	4489(73.0)	
Smoking			0.465
Yes	2898(41.21)	2572(41.8)	
No	4135(58.79)	3576(58.2)	
Vitamin D deficiency			0.820
Yes	2254(32.05)	1959(31.86)	
No	4779(67.95)	4189(68.14)	
Age,y	48.00(32.00-63.00)	48.00(32.00-63.00)	0.965
Body mass indexb	28.00(24.20-32.60)	28.10(24.30-35.80)	0.233
Serum cotinine (ng/mL)	0.035(0.01-4.83)	0.035(0.01-5.25)	0.999
Cholesterol (mmol/L)	4.81(4.14-5.51)	4.78(4.14-5.51)	0.818
Total Urine Arsenic(μg/L)	6.83(3.48-14.91)	6.58(3.38-14.03)	0.040
Dimethylarsinate(μg/L)	3.41(1.85-6.01)	3.32(1.35-5.81)	0.022
